# Effects of Transdiagnostic Cognitive Behavioural Therapy on Long‐Term Quality of Life: A Causal Mediation Analysis Across Anxiety and Depressive Symptoms

**DOI:** 10.1155/da/1601969

**Published:** 2026-02-06

**Authors:** Gabriel Esteller-Collado, María Carpallo-González, Maider Prieto-Vila, Francisco Jurado-González, Mario Gálvez-Lara, Paloma Ruíz-Rodríguez, César González-Blanch, Juan Antonio Moriana, Antonio Cano-Vindel, Roger Muñoz-Navarro

**Affiliations:** ^1^ Department of Personality, Assessment and Psychological Treatments, Faculty of Psychology, Universitat De València, Valencia, Spain, upv.es; ^2^ Department of Psychology, Faculty of Biomedical and Health Sciences, Universidad Europea De Madrid, Madrid, Spain, uem.es; ^3^ Department of Psychology, Faculty of Psychology, University of Cordoba, Cordoba, Spain, uco.es; ^4^ Maimonides Biomedical Research Institute of Cordoba (IMBIC), Cordoba, Spain; ^5^ Reina Sofía University Hospital, Cordoba, Spain, juntadeandalucia.es; ^6^ Tres Cantos Primary Care Centre, Health Service of Madrid, Madrid, Spain; ^7^ Department of Psychology, International University of La Rioja (UNIR), Logroño, Spain, unir.net; ^8^ Mental Health Centre, Marqués De Valdecilla University Hospital-IDIVAL, Santander, Spain, humv.es; ^9^ Department of Experimental Psychology, Cognitive Processes and Speech Therapy, Faculty of Psychology, Complutense University of Madrid, Madrid, Spain, ucm.es

**Keywords:** anxiety, depression, quality of life, serial mediation analyses, transdiagnostic cognitive behaviour therapy

## Abstract

**Introduction:**

Anxiety and depression significantly impair quality of life (QoL) in primary care (PC) patients. While transdiagnostic cognitive behavioural therapy (TD‐CBT) is effective, the mechanisms underlying its long‐term impact on QoL remain unclear. This study examined whether changes in anxiety and depressive symptoms mediate the effect of TD‐CBT on QoL at 12‐month follow‐up.

**Methods:**

Data were used from the “Psychology in Primary Care” trial (PsicAP, from its Spanish acronym), which included 1061 PC patients with anxiety and depression, randomised to TD‐CBT plus treatment‐as‐usual (TAU) or TAU alone. Anxiety and depression were assessed at baseline, post‐treatment and 6‐month follow‐up. QoL was measured at baseline and 12‐month follow‐up. Path analyses using structural equation modelling (SEM) were used to study direct and indirect effects, controlling for baseline scores and gender.

**Results:**

TD‐CBT significantly reduced anxiety and depression immediately post‐treatment compared to TAU. The only significant indirect effect on 12‐month QoL across all dimensions operated sequentially through sustained reductions in depressive symptoms. No significant mediation was found via anxiety symptoms. No specific temporal sequence of symptom improvement mediating QoL was identified.

**Discussion:**

TD‐CBT improves anxiety and depressive symptoms at post‐treatment; nevertheless, long‐term QoL improvement occurs primarily through sustained reduction in depressive symptoms and through the direct effects of treatment itself. These findings support the implementation of TD‐CBT in PC to achieve lasting functional recovery, highlighting the crucial role of addressing and sustaining improvements in depression.

## 1. Introduction

Anxiety and depression disorders are the most prevalent mental health problems worldwide [1] and represent a significant burden with regard to health, disability and quality of life (QoL) loss [2]. In most countries, these disorders are treated in the primary care (PC) setting [3]. Nevertheless, the identification and treatment of these disorders still pose a significant challenge [4], as a considerable proportion of patients do not receive adequate assessment and intervention due to various barriers, such as limited access to mental health specialists or lack of resources in public health services [5].

Cognitive behavioural therapy (CBT) is the recommended first‐line treatment for these disorders[6, 7] as it has been shown to be more effective than treatment‐as‐usual (TAU), which is primarily based on the use of psychotropic drugs and/or medical advice, both in the short and long term[8]. However, the implementation of CBT in PC settings is encumbered by several challenges, including the necessity for specialised training, the extended duration of diagnostic protocols and the complexity of treating patients with comorbidities [9]. In light of these challenges, transdiagnostic CBT (TD‐CBT) has emerged as a promising alternative. Unlike traditional diagnosis‐specific CBT, which focuses on distinct protocols for specific symptoms, TD‐CBT targets shared psychological mechanisms underlying multiple disorders—such as emotional dysregulation and repetitive negative thinking [10, 11]—by applying unified therapeutic strategies like cognitive restructuring [12, 13].

While the efficacy of TD‐CBT in treating these disorders is well documented [8, 14] more research is still needed to better understand the underlying processes through which it achieves sustained improvements in overall well‐being over the long term. A particularly relevant outcome in this context is QoL, a multidimensional construct that reflects an individual’s subjective perception of his or her position in life, in the context of the culture and value systems in which he or she lives and in relation to his or her goals and expectations [15, 16]. QoL comprises several facets of well‐being, including psychological, physical, social and environmental dimensions [17]. Improving QoL is a central goal of treatment, as it indicates functional recovery beyond simple symptomatic remission [18–20].

Previous research has suggested that anxiety and depression affect all dimensions of QoL, but their impact may differ in terms of magnitude and time course [21–23]. Anxiety has been more strongly associated with physical QoL due to somatic symptoms such as muscle tension, fatigue and cardiovascular problems, whereas depression has a more profound effect on psychological and social QoL by influencing mood, motivation and cognitive processing [22, 24]. Furthermore, although both symptoms negatively affect QoL, depressive symptoms have often been found to exert a more pronounced and pervasive impact on QoL compared to anxiety symptoms, especially when both disorders co‐occur, which is common in the PC setting [25].

Despite this relationship between symptoms and QoL, it remains unclear whether the effects of TD‐CBT occur simultaneously or whether there is a specific sequence in which changes in symptoms influence QoL. Some theoretical models propose that improvements in anxiety may occur earlier than improvements in depression, given that anxiety tends to manifest more reactively and with more overt physiological symptoms, whereas depression tends to be more stable and resistant to change [26, 27]. From a clinical perspective, this suggests that interventions such as TD‐CBT might initially target anxiety symptoms, subsequently facilitating reductions in depression and ultimately leading to improvements in QoL [28]. However, this hypothesis has been little explored in longitudinal studies with robust methodologies, such as path analyses using structural equation modelling (SEM).

The present study aimed to examine in a sample of PC patients how changes in anxiety and depressive symptoms, assessed at post‐treatment and at 6‐month follow‐up, act as a pathway of change through which TD‐CBT improves different dimensions of QoL at 12‐month follow‐up. Additionally, we sought to explore the temporal interrelationships between the improvement of anxiety and depression symptoms within this process of change towards better QoL in the long term.

## 2. Methods

### 2.1. Participants and Procedure

Data from the longitudinal randomised clinical trial (RCT) PsicAP [14], conducted in 22 PC centres of the Spanish National Health System, were used. General practitioners (GP) at the participating centres invited all patients aged 18–65 years with a diagnosis (or suspected diagnosis) of an emotional disorder (anxiety, depression or somatizations) to participate in the trial. The patients were screened and assessed to determine whether they met the study inclusion criteria, which was a score above the cut‐off points on at least one of the subscales of the Generalised Anxiety Disorder‐7 (GAD‐7 ≥ 10 points), Patient Health Questionnaire‐9 (PHQ‐9 ≥ 10 points), or PHQ‐15 (PHQ‐15 ≥ 10 points). Exclusion criteria were the presence of any of the following: severe depressive symptoms (PHQ‐9 ≥ 24); high level of functional disability (Sheehan Disability Scale ≥ 26); recent suicide attempt; currently receiving psychological treatment; substance dependence disorder; or severe psychological disorder (personality, eating, bipolar or psychotic disorder). The exclusion for major depressive symptoms is due to the fact that the treatment protocol dispensed was designed for the treatment of mild and moderate symptoms. More information on the treatment protocol and study design can be found in Supporting Information 3: Table S3 and the original trial protocol and subsequent publications [29, 30].

A total of 1061 patients were included in the PsicAP trial and randomised to either the control arm (TAU; *n* = 534) or the intervention arm (TD‐CBT + TAU; *n* = 527). Of these patients, 623 completed the post‐treatment measures (TAU = 313 and TD‐CBT + TAU = 310), 432 completed the 6‐month follow‐ups (TAU = 204 and TD‐CBT + TAU = 228) and 388 completed the 12‐month follow‐ups (TAU = 180 and TD‐CBT + TAU = 208). Table 1 shows the socio‐demographic characteristics of all patients included in the trial.

**Table 1:** Socio‐demographic characteristics of the sample.

Characteristics	Total (*n* = 1061)	TD‐CBT + TAU (*n* = 527)	TAU (*n* = 534)
Gender			
Female	861 (81.1)	424 (80.5)	437 (81.8)
Male	200 (18.9)	103 (19.5)	97 (18.2)
Age	42.9 (11.8)	42.7 (11.6)	43.1 (12.0)
Age group			
≤19	16 (1.5)	10 (1.9)	6 (1.1)
20–39	386 (36.4)	189 (35.9)	197 (36.9)
40–59	581 (54.8)	295 (56.0)	286 (53.6)
≥60	78 (7.4)	33 (6.3)	45 (8.4)
Marital status			
Married	513 (48.4)	265 (50.3)	248 (46.4)
Divorced	87 (8.2)	53 (10.1)	34 (6.5)
Widowed	29 (2.7)	15 (2.8)	14 (2.6)
Separated	58 (5.5)	21 (4.0)	37 (6.9)
In couple	212 (20.0)	110 (20.0)	102 (19.1)
Unmarried	162 (15.3)	63 (12.0)	99 (18.5)
Level of education			
No schooling	11 (1.0)	4 (0.8)	7 (1.3)
Basic education	267 (25.2)	127 (24.1)	140 (269.2)
Secondary education	233 (22.0)	111 (21.1)	122 (22.8)
High school	262 (24.7)	139 (26.4)	123 (23.0)
Bachelor	242 (22.8)	123 (23.3)	119 (22.3)
Master/doctorate	46 (4.3)	23 (4.4)	23 (4.3)
Employment situation			
Full time	248 (14.7)	93 (17.6)	87 (16.3)
Part time	633 (37.4)	183 (34.7)	209 (39.1)
Unemployed	568 (33.5)	184 (34.9)	183 (34.2)
Temp. incapacity	129 (7.6)	41 (7.8)	32 (6.0)
Perm. incapacity	37 (2.2)	13 (2.5)	10 (1.9)
Retired	76 (4.5)	13 (2.5)	13 (2.4)
Level of income			
Less than 12.000 €	670 (39.6)	195 (37.0)	214 (40.0)
12.000 € ‐ 24.000 €	690 (40.8)	218 (41.4)	215 (40.2)
24.000 € ‐ 36.000 €	218 (12.9)	73 (13.9)	74 (13.9)
More than 36.000 €	113 (6.7)	41 (7.8)	31 (5.8)

*Note*: age and age group expressed in years; in all characteristics: *n* (%), except age: *M* (*SD*).

Abbreviations: ITT, intention to treat; PP, per protocol; TAU, treatment as usual; TD‐CBT, transdiagnostic cognitive behavioural therapy.

The intervention was delivered by trained clinical psychologists and consisted of seven group sessions (8–10 patients) over a 12–14 week period. The therapeutic approach was transdiagnostic. The study protocol is described in detail elsewhere [29]. In the TAU arm, the treatment was left to the discretion of the participating GPs but mainly consisted of psychotropic drugs and medical advice.

### 2.2. Instruments

#### 2.2.1. Measures of Symptoms

##### 2.2.1.1. PHQ‐9

The PHQ‐9 was used to assess depressive symptoms experienced during the last 2 weeks [31]. The PHQ‐9 is a nine‐item scale based on DSM‐IV criteria for major depression. Responses are given on a four‐point Likert‐type scale (0 = never; 3 = almost every day), with a maximum score of 27 points. This questionnaire has good psychometric properties and has been validated in a sample of PC patients in Spain [32]. The reliability was good (*α* = 0.86).

##### 2.2.1.2. GAD‐7

The GAD‐7 was used to assess symptoms of generalised anxiety experienced during the last 2 weeks [33]. The questionnaire consists of seven items with responses given on a four‐point Likert‐type scale (0 = never; 3 = almost every day). The maximum score is 21 points. Responses are based on the frequency of symptoms in the last 2 weeks. We used the Spanish version validated by Garcia‐Campayo et al. [34], which has also been specifically validated for use in the PC setting [35, 36], with good psychometric properties. The internal consistency of the instrument was good (*α* = 0.87).

#### 2.2.2. Measures of QoL

##### 2.2.2.1. World Health Organization QoL Instrument‐Abbreviated Version (WHOQOL‐Bref)

The WHOQOL‐Bref was used to assess QoL [37]. This instrument is an abbreviated version of the original WHOQOL, with 26 items rated on a five‐point Likert‐type response scale (1 = not at all; 5 = completely). This questionnaire provides a score on four specific dimensions: psychological, physical, social and environmental. A higher score indicates a higher level of QoL. All dimensions showed acceptable reliability in the sample, psychological QoL (*α* = 0.79), physical QoL (*α* = 0.77), social QoL (*α* = 0.70) and environmental QoL (*α* = 0.79).

### 2.3. Statistical Analysis

The analyses conducted in this study were based on the statistical paradigm of mediation [38]. The main objective of mediation is to explain ’how’ one variable exerts a causal effect on another through one or more intermediary variables, known as mediators. In order to answer the research hypotheses, four path analysis models were conducted within the SEM framework, one for each dimension of QoL (psychological, physical, social and environmental). Four separate models were run to facilitate interpretation of the results and to avoid over‐parameterisation that would result from a single model with all dimensions included simultaneously [39]. Furthermore, given the correlation between the different dimensions of QoL (*r* = 0.43–0.61) separate analyses of each dimension could provide relevant specific information.

**Figure 1:**
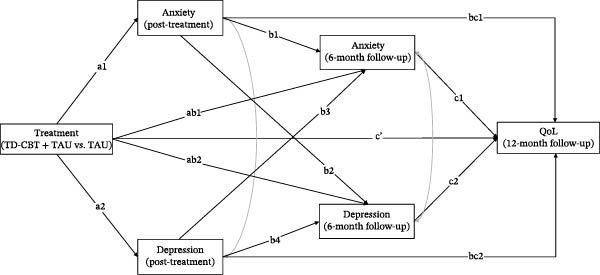
Hypothetical model of path analysis models proposed. Note: anxiety and depression symptoms are measured after treatment and at 6‐month follow‐up. QoL dimensions are measured in 12‐month follow‐up. Treatment (TD‐CBT + TAU vs. TAU). Pretreatment anxiety and depression scores were entered as covariates at post‐treatment and 6‐month follow‐up time points. Pretreatment scores on QoL were entered as a covariate at the 12‐month follow‐up time point. Gender was also entered as a covariate at all time points. Covariance between anxiety and depressive symptoms was controlled for at post‐treatment and at 6‐month follow‐up.

In all models, the independent variable was the treatment received (TD‐CBT + TAU vs. TAU), coded as TD‐CBT + TAU = 1 and TAU = 0. The mediating variables were anxiety (GAD‐7) and depression (PHQ‐9) symptoms measured at post‐treatment assessment and at 6‐month follow‐up. Autoregressive pathways were included to clarify whether treatment‐induced change in symptoms explains the observed differences in the different dimensions of QoL. Furthermore, this design allows for the study of crossover effects between time points to study the possible sequence of change among these symptoms. The dependent variable was QoL measured at 12 months follow‐up (in each model a different dimension). In all models we controlled for covariance between anxiety and depressive symptoms at post‐treatment and 6‐month follow‐up. Pretreatment anxiety, depression and QoL scores were also entered as covariates at all time points. In addition, given the difference in the proportion of men and women in the sample, gender was also entered as a covariate (Figure 1).

Fit was assessed for all models using the comparative fit index (CFI), the Tucker–Lewis Index (TLI), the root mean square error of approximation (RMSEA) and the standardised root mean square residual (SRMR). Values above 0.95 for the CFI and TLI were considered to reflect a good model fit. RMSEA and SRMR values of 0.09 or less were considered to indicate a good fit [39, 40]. Since there was a considerable amount of missing data, the full information maximum likelihood (FIML) estimator was used, as it is a robust alternative that uses all available information without the need for prior imputations. FIML estimates parameters by maximising the likelihood based on the observed data, which makes it an efficient and recommended method to handle missing values adequately in SEM.

In all of these models, we evaluated the direct effects between the variables, the indirect effects and the total effect (*c*). The indirect effect was considered significant if the calculated confidence interval did not include the value 0. In all models, 1000 bootstrapping samples were used to build the confidence intervals. All effects are reported as standardised *β* values. Figure 1 shows the theoretical hypothetical model of the different models.

Data management and analyses were carried out using Statistical Package for Social Sciences (SPSS v.29) and *R* (4.3.3) software. SEM models were estimated using the lavaan package.

## 3. Results

### 3.1. Descriptive Statistics

The sample had a mean age of 43.6 years (*SD* = 12.3) and was predominantly female (81.1%). The majority were married (48.4%) or in a couple (20.0%), employed part‐time (37.4%) or unemployed (33.5%), and had an income level of 24.000 euros/year or less (80.4%). Symptom scores for anxiety and depression, as well as QoL dimensions, did not differ between groups (TD‐CBT + TAU vs. TAU) at pretreatment (Table 2). Table 2 also summarises the means (*SD*) of the variables involved in the analyses, both at post‐treatment and at 6‐month follow‐up for symptoms, and at 12‐month follow‐up for QoL dimensions. Due to the high dropout rate in the sample, and to ensure generalisability of the results, supplementary analyses were performed between the treated sample and the non‐treated sample. Supporting Information 1: Table S1 and Supporting Information 2: Table S2 show that treatment dropout was not related to treatment allocation condition (TD‐CBT + TAU vs. TAU) nor to symptomatic severity.

**Table 2:** Baseline and time‐dependent differences for all study variables.

Outcome	TD‐CBT + TAU		TAU		Difference
	*N*	*M* (SD)	*N*	*M* (SD)	*p*
PHQ‐9					
Baseline	527	13.7 (5.3)	534	13.5 (5.1)	0.443
Post‐treatment	310	7.0 (5.2)	313	11.5 (6.6)	<0.001
6‐month FU	228	7.2 (6.1)	204	10.0 (6.5)	<0.001
GAD‐7					
Baseline	527	12.4 (4.6)	534	12.1 (4.6)	0.264
Post‐treatment	310	6.0 (4.3)	313	10.2 (5.5)	<0.001
6‐month FU	228	6.1 (4.8)	204	8.8 (5.6)	<0.001
Psychological QoL					
Baseline	527	16.7 (3.8)	534	16.8 (3.8)	0.578
12‐month FU	208	20.2 (4.6)	180	18.3 (4.4)	<0.001
*Physical QoL*					
Baseline	527	22.1 (4.3)	534	22.4 (4.2)	0.327
12‐month FU	208	25.6 (5.3)	180	22.7 (5.1)	<0.001
					
Social QoL					
Baseline	527	9.0 (2.4)	534	9.0 (2.4)	0.472
12‐month FU	208	10.0 (2.5)	180	9.3 (2.2)	<0.001
					
Environmental QoL					
Baseline	527	25.2 (4.5)	534	25.6 (4.6)	0.138
12‐month FU	208	28.3 (5.3)	180	26.5 (5.0)	<0.001

Abbreviations: FU, follow‐up; GAD‐7, generalized anxiety disorder‐7; PHQ‐9, patient health questionnaire‐9; QoL, quality of life; TAU, treatment as usual; TD‐CBT, transdiagnostic cognitive behavioural therapy.

### 3.2. Path Analyses

All models estimated showed a good fit (Table 3). Although *χ* ^2^ is significant, the fit can be considered adequate, as *χ* ^2^ is particularly sensitive to sample size and all other indices are within acceptable ranges. In all models the CFI is >0.98 and the TLI >0.93, the RMSEA <0.08 and the SRMR is <0.07.

**Table 3:** Summary of modelling adjustment.

Model	*χ* ^2^	*p*	CFI	TLI	RMSEA	SRMR
Psychological QoL	48.208	< 0.001	0.984	0.945	0.060	0.066
Physical QoL	53.536	< 0.001	0.981	0.932	0.063	0.061
Social QoL	35.241	< 0.001	0.988	0.959	0.049	0.047
Environmental QoL	43.610	< 0.001	0.986	0.952	0.056	0.060

*Note: χ* ^2^: Chi‐square; *p*: signification.

Abbreviations: CFI, comparative fit index; QoL, quality of life; RMSEA, root mean square error of approximation; SRMR, standarized root mean square; TLI, Tucker–Lewis Index.

**Figure 2:**
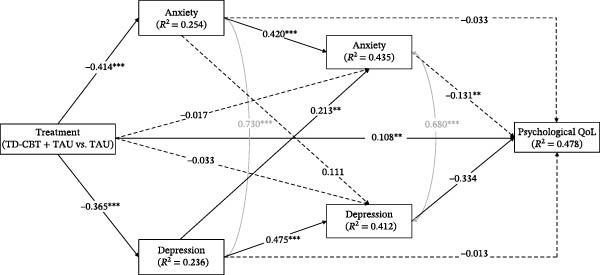
Direct and total effects of psychological QoL model. Note:  ^∗^ *p* < .05;  ^∗∗^ *p* < .01;  ^∗∗∗^ *p* < .001. Anxiety and depression symptoms are measured after treatment and at 6‐month follow‐up. QoL dimensions are measured in 12‐month follow‐up. Treatment (TD‐CBT + TAU vs. TAU). Pretreatment anxiety and depression scores were entered as covariates at post‐treatment and 6‐month follow‐up time points. Pretreatment scores on psychological QoL were entered as a covariate at the 12‐month follow‐up time point. Gender was also entered as a covariate at all time points. Covariance between anxiety and depressive symptoms was controlled for at post‐treatment and at 6‐month follow‐up.

**Figure 3:**
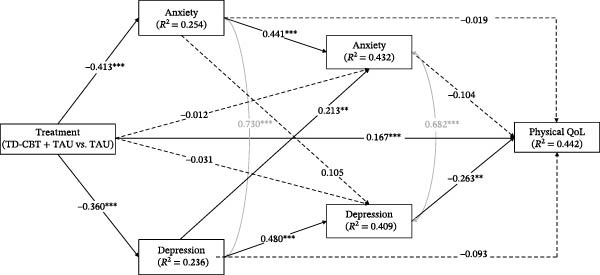
Direct and total effects of physical QoL model. Note:  ^∗^ *p* < .05;  ^∗∗^ *p* < .01;  ^∗∗∗^ *p* < .001. Anxiety and depression symptoms are measured after treatment and at 6‐month follow‐up. QoL dimensions are measured in 12‐month follow‐up. Treatment (TD‐CBT + TAU vs. TAU). Pretreatment anxiety and depression scores were entered as covariates at post‐treatment and 6‐month follow‐up time points. Pretreatment scores on physical QoL were entered as a covariate at the 12‐month follow‐up time point. Gender was also entered as a covariate at all time points. Covariance between anxiety and depressive symptoms was controlled for at post‐treatment and at 6‐month follow‐up.

**Figure 4:**
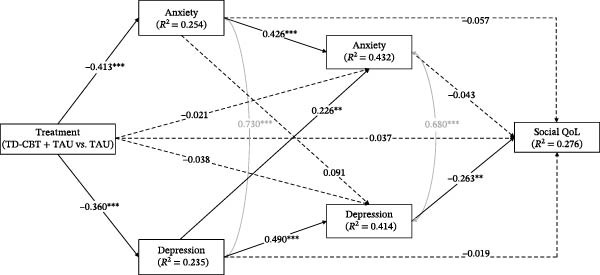
Direct and total effects of the social QoL model. Note: ^∗^ *p* < .05;  ^∗∗^ *p* < .01;  ^∗∗∗^ *p* < .001. Anxiety and depression symptoms are measured after treatment and at 6‐month follow‐up. QoL dimensions are measured in 12‐month follow‐up. Treatment (TD‐CBT + TAU vs. TAU). Pretreatment anxiety and depression scores were entered as covariates at post‐treatment and 6‐month follow‐up time points. Pretreatment scores on social QoL were entered as a covariate at the 12‐month follow‐up time point. Gender was also entered as a covariate at all time points. Covariance between anxiety and depressive symptoms was controlled for at post‐treatment and at 6‐month follow‐up.

**Figure 5:**
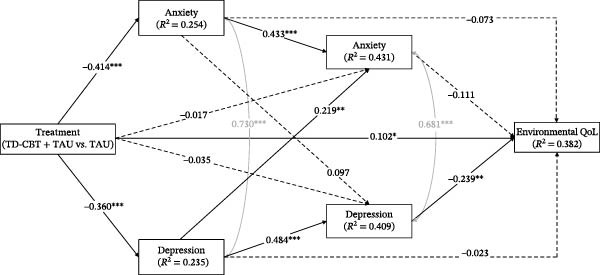
Direct and total effects of environmental QoL model. Note:  ^∗^ *p* < .05;  ^∗∗^ *p* < .01;  ^∗∗∗^ *p* < .001. Anxiety and depression symptoms are measured after treatment and at 6‐month follow‐up. QoL dimensions are measured in 12‐month follow‐up. Treatment (TD‐CBT + TAU vs. TAU). Pretreatment anxiety and depression scores were entered as covariates at post‐treatment and 6‐month follow‐up time points. Pretreatment scores on environmental QoL were entered as a covariate at the 12‐month follow‐up time point. Gender was also entered as a covariate at all time points. Covariance between anxiety and depressive symptoms was controlled for at post‐treatment and at 6‐month follow‐up.

In all models, treatment (in favour of TD‐CBT + TAU) showed significant direct effects on anxiety and depressive symptoms at post‐treatment, as well as on QoL at 12 months. Treatment had no significant direct effect on anxiety and depressive symptoms at 6‐month follow‐up. Anxiety symptoms at post‐treatment had significant direct effects on anxiety symptoms themselves at 6 months but not on depression symptoms at 6 months and QoL at 12 months. Depressive symptoms had significant direct effects on own depressive symptoms and anxiety symptoms at 6 months, but not on QoL at 12 months. Finally, only depressive symptoms at 6 months had a significant direct effect on QoL at 12 months follow‐up (Figure 2, 3, 4 and 5). Gender, which was entered as a covariate in all regressions of the models, was not significant at any time point.

In terms of percentage of variance explained, all models explained approximately: 25% of anxiety symptoms and 23% of depression symptoms at post‐treatment; 43% of anxiety symptoms and 41% of depression symptoms at 6‐month follow‐up; and 48% of psychological QoL, 44% of physical QoL, 28% of social QoL and 38% of environmental QoL.

Finally, in terms of indirect effects, the only one that was significant in all models was the one through the whole sequence of depressive symptoms (indirect effect 6, Table 4). No other partial indirect effects through depression or through anxiety were significant. The total treatment effect on the different dimensions of QoL was also significant in all models.

**Table 4:** Indirect and total effects of path analysis models on QoL dimensions.

Model	*β*	SE	95% CI
Psychological QoL			
(Ind_1_) a_1_‐bc_1_	0.016	0.259	[−0.30 – 0.68]
(Ind_2_) a_2_‐bc_2_	0.005	0.282	[−0.55 – 0.69]
(Ind_3_) ab_1_‐c_1_	0.002	0.057	[−0.09 – 0.15]
(Ind_4_) ab_2_‐c_2_	0.011	0.144	[−0.14 – 0.45]
(Ind_5_) a_1_‐b_1_‐c_1_	0.024	0.142	[−0.08 – 0.53]
(Ind_6_) a_2_‐b_4_‐c_2_	**0.057**	**0.154**	**[0.21 – 0.86]**
(Ind_7_) a_1_‐b_2_‐c_2_	0.015	0.106	[−0.02 – 0.40]
(Ind_8_) a_2_‐b_3_‐c_1_	0.010	0.056	[−0.01 – 0.21]
Total effect	0.248	0.382	[1.58 – 2.96]
Physical QoL			
(Ind_1_) a_1_‐bc_1_	0.008	0.344	[‐0.83 – 0.60]
(Ind_2_) a_2_‐bc_2_	0.033	0.352	[‐0.19 – 0.32]
(Ind_3_) ab_1_‐c_1_	0.002	0.063	[‐0.10 – 0.16]
(Ind_4_) ab_2_‐c_2_	0.008	0.115	[‐0.16 – 0.31]
(Ind_5_) a_1_‐b_1_‐c_1_	0.019	0.191	[‐0.10 – 0.63]
(Ind_6_) a_2_‐b_4_‐c_2_	**0.046**	**0.212**	**[0.07 – 0.94]**
(Ind_7_) a_1_‐b_2_‐c_2_	0.011	0.110	[−0.06 – 0.41]
(Ind_8_) a_2_‐b_3_‐c_1_	0.008	0.076	[−0.05 – 0.25]
Total effect	0.286	0.413	[2.30 – 3.97]
Social QoL			
(Ind_1_) a_1_‐bc_1_	0.023	0.181	[−0.25 – 0.43]
(Ind_2_) a_2_‐bc_2_	0.007	0.155	[−0.24 – 0.33]
(Ind_3_) ab_1_‐c_1_	0.001	0.021	[−0.03 – 0.05]
(Ind_4_) ab_2_‐c_2_	0.010	0.055	[−0.05 – 0.18]
(Ind_5_) a_1_‐b_1_‐c_1_	0.008	0.079	[−0.13 – 0.20]
(Ind_6_) a_2_‐b_4_‐c_2_	**0.045**	**0.089**	**[0.08 – 0.40]**
(Ind_7_) a_1_‐b_2_‐c_2_	0.010	0.046	[−0.03 – 0.15]
(Ind_8_) a_2_‐b_3_‐c_1_	0.004	0.033	[−0.06 – 0.08]
Total effect	0.145	0.196	[0.28 – 1.10]
Environmental QoL			
(Ind_1_) a_1_‐bc_1_	0.030	0.301	[−0.87 – 0.27]
(Ind_2_) a_2_‐bc_2_	0.008	0.308	[−0.55 – 0.63]
(Ind_3_) ab_1_‐c_1_	0.002	0.068	[−0.07 – 0.23]
(Ind_4_) ab_2_‐c_2_	0.008	0.127	[−0.14 – 0.41]
(Ind_5_) a_1_‐b_1_‐c_1_	0.020	0.165	[−0.11 – 0.52]
(Ind_6_) a_2_‐b_4_‐c_2_	**0.042**	**0.177**	**[0.12 – 0.81]**
(Ind_7_) a_1_‐b_2_‐c_2_	0.010	0.093	[−0.07 – 0.32]
(Ind_8_) a_2_‐b_3_‐c_1_	0.009	0.081	[−0.04 – 0.29]
Total effect	0.170	0.449	[0.83 – 2.75]

*Note:* The indirect effect is statistically significant when the confidence interval (CI) does not include the value zero. Significant indirect effects are marked in bold. Standard errors refer to non‐standardised betas. *β*, standardised beta coefficient.

Abbreviations: CI, confidence interval; QoL, quality of life. SE, standard error.

## 4. Discussion

The main aim of this study was to examine whether change in anxiety and depressive symptoms is a mechanism underlying the efficacy of TD‐CBT in improving QoL in PC patients. Specifically, we investigated whether changes in anxiety and depression symptoms, assessed at post‐treatment and at 6‐month follow‐up, mediated the relationship between treatment and different QoL dimensions at 12‐months follow‐up. Additionally, temporal interrelationships between the improvement of anxiety and depressive symptoms in this process of change were explored. Results showed that TD‐CBT had a significant direct effect on the reduction of anxiety and depressive symptoms at post‐treatment, but not on symptom changes at 6 months. In all models, the only significant indirect effect was that of operating across the entire sequence of depressive symptom reduction. That is, TD‐CBT reduced post‐treatment depression, this reduction influenced change at 6 months, and these changes in turn positively influenced QoL at 12 months. No significant mediation effects involving only anxiety or partial indirect effects were found. Contrary to expectations, no time‐specific sequence of action of TD‐CBT on anxiety and depressive symptoms was found.

In all models, direct treatment effects on anxiety and depressive symptoms were significant in favour of TD‐CBT + TAU. These results are consistent with previous studies that have shown TD‐CBT to be an effective treatment approach for treating anxiety disorders and depression [8, 41]. Some work has previously sought to determine why transdiagnostic approaches are particularly effective for treating these disorders [13, 42, 43]. One explanation is based on the common aetiology of these disorders and their high rates of comorbidity. As noted by Newby et al. [44], dual diagnosis of depression and anxiety is more common in the PC setting than single diagnosis. A second explanation is that transdiagnostic treatments address change processes that are common to different disorders, such as exposure strategies, behavioural activation or emotional regulation [42, 45]. However, it is remarkable that this direct treatment effect was not significant for symptoms assessed at 6‐month follow‐up. This absence of a medium‐term effect suggests that the main causal influence of the intervention is concentrated in the immediate post‐treatment period. The improvement observed subsequently appears to depend more on the stability of the initial changes achieved, reflected through autoregressive symptom pathways [39, 46–48].

A relevant finding of this study is the direct and positive effect of TD‐CBT on the psychological, physical and environmental QoL dimensions at 12 months. This suggests that TD‐CBT may promote long‐term well‐being through mechanisms other than simply reducing symptoms of anxiety and depression. It is plausible that core components of the intervention, such as cognitive restructuring, behavioural activation or training in emotional regulation [14, 30], directly impact self‐efficacy, coping strategies or perceived control over one’s own physical and mental health, key factors in these dimensions of QoL [17]. Conversely, the absence of this direct effect on the social dimension of QoL is remarkable, thus generating a total mediation effect through symptoms. A possible explanation for this phenomenon could be that social functioning and satisfaction are often profoundly affected by anxious–depressive symptomatology (e.g., social avoidance, anhedonia and isolation) [27, 49, 50], so that substantial improvements in this area may be more closely dependent on significant and sustained symptomatic remission. Furthermore, this dimension of QoL may also be influenced by more stable contextual factors (e.g., pre‐existing support network) that are less amenable to direct modification by brief psychological intervention [51].

In terms of the mechanisms of change underlying the treatment effect on QoL, the most striking finding was the identification of a single significant and consistent indirect pathway across all models. This pathway operated through a sequence in which TD‐CBT reduced post‐treatment depression, this initial improvement favoured the maintenance of low levels of depression at 6 months, and it was these reduced levels at 6 months that ultimately predicted better QoL at 12 months. This result is consistent with the literature highlighting the particularly profound and pervasive impact that depressive symptomatology exerts on multiple functional domains and subjective perception of QoL, often more pervasively than anxiety [22, 25]. The presence of this full sequence, which requires maintenance of depressive improvement up to 6 months, suggests that an initial reduction is not sufficient, but that it is the consolidation of such improvement that translates robustly into lasting benefits in QoL, considered an essential indicator of functional recovery [18, 48, 52].

In contrast, although TD‐CBT also induced a significant reduction in anxiety at post‐treatment, our analyses did not support a mediating role for changes in anxiety symptoms in explaining the improvement in QoL at 12 months. Other indirect pathways that did not involve the full sequence through depression were also not significant. These findings have important implications for transdiagnostic theory. While TD‐CBT focuses on the common core of negative affectivity, the path to functional recovery appears to be different for each disorder. Anxiety, characterised by hyperarousal and sensitivity to threats, may be more situational and somatic, acting as an ‘alarm’ that fluctuates without necessarily defining long‐term life satisfaction [24]. In contrast, depressive symptoms, particularly anhedonia and hopelessness, directly erode the capacity for enjoyment and purpose, which are central to the concept of QoL [25]. Furthermore, overlapping measures are likely to reinforce this effect; PHQ‐9 items (e.g., fatigue and concentration) reflect functional domains of QoL more closely than GAD‐7 arousal symptoms, resulting in stronger statistical covariance [22, 53]. Likewise, the nature of some anxiety symptoms, which may be more reactive to situational stressors or have a greater physiological component, may also contribute to a less stable or direct relationship with long‐termQoL compared to the affective–cognitive core of depression [54].

Finally, it is important to note that no specific and preferential temporal sequence was identified in which TD‐CBT mediated one type of symptom before the other to influence QoL. That is, neither the pathway involving an initial reduction in anxiety followed by changes in depression nor the reverse pathway, was significant as an explanatory mechanisms for change in QoL. This absence of a defined temporal sequence of action may reflect the highly interrelated nature and reciprocal influence that characterises anxiety and depression symptoms, especially in clinical samples with high comorbidity such as PC [3]. It is also consistent with the philosophy of TD‐CBT itself, which postulates and addresses common transdiagnostic mechanisms (e.g., emotional regulation, attentional processes or negative thinking patterns) whose improvement could translate into relatively simultaneous or interdependent reductions in both types of symptoms, rather than following a fixed sequential cascade [42, 43]. In addition, individual heterogeneity in treatment responses or the measurement intervals used (post‐treatment and 6 months) might have made it difficult to detect more rapid or specific sequences at the subgroup level. Taken together, these results support a view of dynamic interdependence between anxiety and depression in the process of change induced by TD‐CBT, rather than a strict sequential causal relationship on the path to QoL improvement.

### 4.1. Clinical Implications

These results have some clinical implications for PC settings. First, they reinforce the value of TD‐CBT not only for acute symptom reduction but also for promoting long‐term improvements in overall QoL, a crucial outcome for patients. And second, the critical role of sustained depressive symptom reduction as a primary mediator underscores the importance of closely monitoring depressive symptoms after the active treatment phase and potentially implementing strategies focused on maintaining these gains to ensure lasting gains in QoL.

### 4.2. Limitations

The present study has some limitations that are important to note. First, all the instruments used to measure symptomatic variables and QoL are self‐report, which may lead to biased and inaccurate results. However, all the measures used in this article have been previously validated in the PC context, showing that they can be valid and reliable clinical indicators of a possible disorder [32, 36]. Second, the long‐term data loss rate was high, with only 388 participants out of the 1061 initially randomised completing follow‐up measures at 12 months. For this reason, the results should be interpreted with caution, as treatment dropout may induce biases in the results. However, in an attempt to mitigate these biases, the FIML estimator was used to fit the SEM models. This procedure has been shown to be a robust alternative to use all the information available in the data without having to perform prior imputations. Finally, the high proportion of female participants (81.1%) reduces the generalisability of the results to the male population, which calls for future research to assess whether mediation sequences and therapeutic effects differ by gender. However, despite this imbalance in the sample, as noted in the results section, gender, controlled for as a covariate in all steps of the models, was not significant at any point.

### 4.3. Future Lines and Conclusions

Future research could conduct longitudinal studies with mid‐term assessments throughout the treatment process (e.g., after each session) to clarify more precisely the temporal and causal sequence of changes in anxiety and depressive symptoms. In addition, it would be valuable to incorporate objective measures, such as standardised clinical assessments, biomarkers or data from digital devices, to complement self‐reports and reduce bias in the measurement of therapeutic response. It would also be interesting to replicate the study in more balanced samples in terms of gender and socio‐demographic diversity, which would allow to analyse possible differences in the efficacy of TD‐CBT in different subgroups. This research will not only deepen the understanding of the underlying mechanisms of change but will also provide key information for the implementation of more personalised and efficient treatments in PC.

In conclusion, this study provides strong evidence for the long‐term efficacy of TD‐CBT in improving QoL among PC patients with anxiety and depression. The results identify sustained reduction in depressive symptoms as a key mechanism driving these improvements, while highlighting the direct beneficial effects of TD‐CBT on well‐being that extend beyond symptom relief. Overall, these results support the implementation of TD‐CBT in PC to achieve meaningful and lasting functional recovery.

## Author Contributions

**Gabriel Esteller-Collado**: conceptualisation, investigation, methodology, formal analysis, visualisation, writing – original draft, writing – review and editing, funding acquisition. **María Carpallo-González**, **Maider Prieto-Vila, Francisco Jurado-González, Mario Gálvez-Lara, Paloma Ruíz-Rodríguez and César González-Blanch:** investigation, writing – review and editing. **Juan Antonio Moriana**: investigation, writing – review and editing, funding acquisition. **Antonio Cano-Vindel**: supervision, project administration, funding acquisition, investigation. **Roger Muñoz-Navarro**: supervision, investigation, writing – review and editing, funding acquisition.

## Funding

This work is part of the projects funded by the MICIU/AEI, through the projects (Grants PID2019‐107243RB‐C21, PID2019‐107243RB‐C22 and CPP2023‐010817). It has also received support from the R&D&I Project (Grant PID2021‐125965OB‐I00), funded by MICIU/AEI and FEDER, EU. It was also a project developed within the framework of the General Collaboration Agreement between the University of Valencia and Banco Santander, S.A., as part of the Santander Postdoctoral Research Grants. Finally, this project has also received support from the José Castillejo (Grant CAS2023/00397) awarded by the MCIN/AEI.

## Disclosure

These entities only provided funding for the recruitment of research staff and played no role in the design of the trial, data collection, analysis or writing of this manuscript.

## Ethics Statement

The study was approved by the National Scientific Research Ethics Committee in Spain, conducted in accordance with the Declaration of Helsinki (EUDRACT: 2013‐001955‐11) and the study protocol was registered (ISRCTN58437086). All participants gave their written informed consent.

## Conflicts of Interest

The authors declare no conflicts of interest.

## Supporting Information

Additional supporting information can be found online in the Supporting Information section.

## Supporting information


**Supporting Information 1** Table S1. Cross tabulation and chi‐square test of treated patients vs. dropouts according to TD‐CBT + TAU or TAU randomisation group.

**Supporting Information 2** Table S2. Mean differences in anxiety and depression symptoms in pretreatment treated patients vs. untreated patients.

**Supporting Information 3** Table S3. Schedule and components of PsicAP protocol.

## Data Availability

The data that support the findings of this study are available from the corresponding author upon reasonable request.
