# Correction to “Effects of Transdiagnostic Cognitive Behavioural Therapy on Long‐Term Quality of Life: A Causal Mediation Analysis Across Anxiety and Depressive Symptoms”

**DOI:** 10.1155/da/9780769

**Published:** 2026-07-29

G. Esteller‐Collado, M. Carpallo‐González, M. Prieto‐Vila, et al., “Effects of Transdiagnostic Cognitive Behavioural Therapy on Long‐Term Quality of Life: A Causal Mediation Analysis Across Anxiety and Depressive Symptoms,” *Depression and Anxiety*, 2026, 1601969, https://doi.org/10.1155/da/1601969.

In the article, there are errors in Figure 2, introduced during manuscript preparation. Specifically:

**Figure 2:**
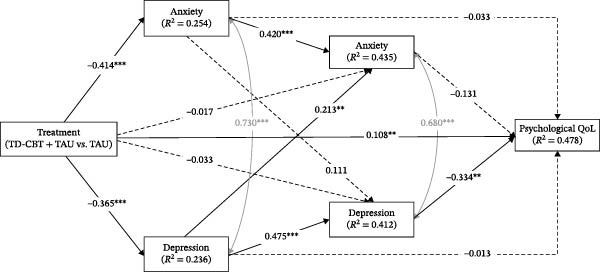
Direct and total effects of psychological QoL model. *Note:*  ^∗^ *p* < 0.05;  ^∗∗^ *p* < 0.01;  ^∗∗∗^ *p* < 0.001. Anxiety and depression symptoms are measured after treatment and at 6‐month follow‐up. QoL dimensions are measured in 12‐month follow‐up. Treatment (TD‐CBT + TAU vs. TAU). Pre‐treatment anxiety and depression scores were entered as covariates at post‐treatment and 6‐month follow‐up time points. Pre‐treatment score on psychological QoL were entered as a covariate at the 12‐month follow‐up time point. Gender was also entered as a covariate at all time points. Covariance between anxiety and depressive symptoms was controlled for at post‐treatment and at 6‐month follow‐up.

• The value associated with the arrow connecting the ‘Anxiety’ box to the ‘Psychological QoL’ box (−1.31) is erroneously marked with asterisks ( ^∗∗^) to indicate significance.
• The value associated with the arrow connecting the ‘Depression’ box to the ‘Psychological QoL’ (−0.334 ^∗∗^) is missing asterisks ( ^∗∗^) to indicate significance.

The correct Figure 2 is shown below:

We apologise for these errors.

