# Supplementary material for: Effects of Transdiagnostic Cognitive Behavioural Therapy on Long‐Term Quality of Life: A Causal Mediation Analysis Across Anxiety and Depressive Symptoms
Source: Depress Anxiety. 2026 Feb 6;2026:1601969. doi: 10.1155/da/1601969 (PMC12881695; doi:10.1155/da/1601969)
Supplement: Supplementary file 1 — Supporting Information 1 Table S1. Cross tabulation and chi‐square test of treated patients vs. dropouts according to TD‐CBT + TAU or TAU randomisation group. [file DA-2026-1601969-s002.docx]

**Supplementary table 1.** Cross-tabulation of treated patients vs dropouts according to randomization group TD-CBT+TAU or TAU.

|  | TD-CBT+TAU (n/%) | TAU (n/%) | Total | χ^2^ | *p* |
| --- | --- | --- | --- | --- | --- |
| Treated | 218 (50.6%) | 212 (49.3%) | 430 | .039 | .843 |
| Dropout | 315 (50.0%) | 314 (49.9%) | 629 |  |  |
| Total | 533 (50.3%) | 526 (49.6%) | 1059 |  |  |

Note: TD-CBT: Transdiagnostic-Cognitive Behavior Therapy; TAU: Treatment as Usual.
