# Supplementary material for: Effects of Transdiagnostic Cognitive Behavioural Therapy on Long‐Term Quality of Life: A Causal Mediation Analysis Across Anxiety and Depressive Symptoms
Source: Depress Anxiety. 2026 Feb 6;2026:1601969. doi: 10.1155/da/1601969 (PMC12881695; doi:10.1155/da/1601969)
Supplement: Supplementary file 2 — Supporting Information 2 Table S2. Mean differences in anxiety and depression symptoms in pretreatment treated patients vs. untreated patients. [file DA-2026-1601969-s001.docx]

**Supplementary table 2.** Mean difference in pre-treatment of treated patients vs non-treated patients.

| Variables | Non-treated patients (M/SD) | Treated patients (M/SD) | *t* | *p* |
| --- | --- | --- | --- | --- |
| Anxiety | 12.2 (4.6) | 12.3 (4.6) | -.336 | .368 |
| Depression | 13.7 (5.1) | 13.6 (5.2) | .262 | .397 |

Note: M: mean; SD: Standard deviation; *t:* t-test effect; *p*: signification; TD-CBT: Transdiagnostic-Cognitive Behavior Therapy; TAU: Treatment as Usual.
