# Supplementary material for: Effects of Transdiagnostic Cognitive Behavioural Therapy on Long‐Term Quality of Life: A Causal Mediation Analysis Across Anxiety and Depressive Symptoms
Source: Depress Anxiety. 2026 Feb 6;2026:1601969. doi: 10.1155/da/1601969 (PMC12881695; doi:10.1155/da/1601969)
Supplement: Supplementary file 3 — Supporting Information 3 Table S3. Schedule and components of PsicAP protocol. [file DA-2026-1601969-s003.docx]

**Supplementary table 3.** Schedule and components of PsicAP protocol

|  | Sessions | | | | | | |
| --- | --- | --- | --- | --- | --- | --- | --- |
| *Psychological techniques* | 1 | 2 | 3 | 4 | 5 | 6 | 7 |
| Psychoeducation | X | X |  |  |  |  |  |
| Relaxation |  | X |  |  |  |  |  |
| Cognitive restructuring |  |  | X | X | X | X | X |
| Behaviour therapy |  |  |  | X | X | X | X |
| Relapse prevention |  |  |  |  |  |  | X |

Note: for more information about study protocol see Cano-Vindel et al. (2016)
